# A Prospective Comparative Study of the Functional Outcomes of Patellar Resurfacing Versus Non-resurfacing in Patients Undergoing Total Knee Arthroplasty

**DOI:** 10.7759/cureus.50380

**Published:** 2023-12-12

**Authors:** Tushar Gogia, Narender Singh, Tirthankar Dasgupta, R Pramod, Dhairya Khera, Nitin Sahu

**Affiliations:** 1 Orthopaedics, Base Hospital Delhi Cantonment, New Delhi, IND; 2 Orthopaedics, Military Hospital Pathankot, Pathankot, IND

**Keywords:** osteoarthritis of knee, patella, anterior knee pain, patellar resurfacing, total knee arthroplasty

## Abstract

Background and objective

Total knee arthroplasty (TKA) is a highly successful surgical procedure. However, there is a lack of consensus about whether to resurface the patella or not. This study was aimed at evaluating the outcome of patellar resurfacing in terms of a decrease in the incidence of anterior knee pain after TKA and assessing whether patellar resurfacing is beneficial in improving functional outcomes.

Materials and methods

This prospective comparative study included 100 patients undergoing TKA who were randomly allotted to the patellar resurfacing or non-resurfacing group. Functional evaluation was done based on the Knee Society Score, and the pain was evaluated by the visual analogue scale (VAS) preoperatively and after one year.

Results

There was a significant improvement in the Knee Society scores as well as the pain scores in both groups postoperatively. The patellar resurfacing group showed statistically significant improvement as compared to the non-resurfacing group in the Knee Society clinical and functional scores as well as the VAS at the end of one year.

Conclusion

Patellar resurfacing during TKA provides better clinical and functional outcomes as well as more relief from anterior knee pain as compared to non-resurfacing of the patella.

## Introduction

Osteoarthritis of the knee is a disabling and painful condition. Total knee arthroplasty (TKA) is a highly successful surgical procedure for knee osteoarthritis [1]. Initially, when total knee prostheses were designed, the patellofemoral articulation was not taken into consideration as a potential source of pain, and the results were affected by patellofemoral symptoms despite an otherwise well-performed knee arthroplasty [1]. High rates of persistent anterior knee pain following early implants, along with other complications such as dislocation, subluxation, and maltracking, were attributed to the patellofemoral joint pathologies. This led to the development of replacements that were tri-compartmental in nature and allowed patellar resurfacing along with femoral and tibial components [2].

Although TKA has commonly been used in severe degenerative osteoarthritis for several years, initially surgeons were not certain about indications of patellar resurfacing. Some authors suggested that patellar resurfacing should be done simultaneously with TKA to relieve anterior knee pain and eliminate the need for a second surgery [3]. Other authors suggested a selective decision based on factors such as the patellar thickness, the presence of preoperative anterior knee pain, the severity of degenerative changes in the patella, the presence of rheumatoid arthritis, and the experience of the surgeon [4].

Progressive degenerative changes of the patella, mainly on the lateral facet, are found as the long-term consequence of the non-resurfacing of the patellofemoral joint in TKA. It was found that 24%-50% of patients who have undergone TKA with an unresurfaced patella experienced anterior knee pain, which reduced the overall impact of TKA from the patient’s perspective. Pain on standing, ascending, or descending stairs and tenderness over the patellofemoral joint were found to be more common in the non-resurfaced group, and routine patellar resurfacing in TKA has been recommended [5, 6].

Resurfacing the patella has certain advantages, like a dramatic reduction in the incidence of post-arthroplasty anterior knee pain and improved strength with fewer needs for revision surgery. However, it has its own set of problems due to poor extensor mechanism kinetics (patellar maltracking) [7-10]. Some studies suggest that patella-related complications were found more often in the resurfaced group than in the group without patellar resurfacing.

However, the controversy about whether to resurface the patella or to leave the native patella unresurfaced continues to be debated by orthopaedic surgeons performing TKA [11].

This study was aimed at evaluating the outcomes of patellar resurfacing in terms of a decrease in anterior knee pain after TKA and assessing whether patellar resurfacing is beneficial in improving functional outcomes.

## Materials and methods

This prospective comparative study was carried out in the department of orthopaedics of Employees State Insurance-Postgraduate Institute of Medical Sciences & Research (ESI-PGIMSR), Basaidarapur, New Delhi, after obtaining clearance from the institutional ethics committee during the years 2020-2022. The sample size of the study was 100 patients. Written informed consent was obtained from all the participants.

A total of 100 patients were selected after either patellar resurfacing or patellar non-resurfacing TKA into the corresponding group using simple random sampling. Standard surgical steps were followed in all the cases, the only difference being the resurfacing of the patella. The same implant design was used in all the cases.

Inclusion and exclusion criteria

Patients aged more than 50 years with osteoarthritis of the knee with less than 30° varus and flexion deformity and significant patellofemoral joint pathology going in for primary TKA were included in the study. Patients with extra-articular deformities, ankylosis, valgus deformities, retained metal hardware around the knee, and revision cases were excluded from the study.

Follow up

The results at the one-year follow-up were compared with the preoperative status of the patients. The results between both groups were compared. The clinical improvement and functionality were assessed through physical examination using the Knee Society clinical scores and functional scores, respectively. The objective of this separation was to ensure that the scores were not affected by variables such as comorbidities and advanced age.

The pain intensity was assessed using the visual analogue scale (VAS), which is the most common way to assess pain.

Statistical analysis

Categorical variables are presented in numbers and percentages (%), and continuous variables are presented as the mean ± SD. The normality of the data has been tested by the Kolmogorov-Smirnov test. Quantitative variables have been compared using the unpaired t-test or Mann-Whitney test (when the data sets were not normally distributed) between the two groups. A paired t-test has been used for intragroup comparison pre-and post-intervention. A p-value less than 0.05 has been considered statistically significant. The data were analysed using IBM Statistical Package for Social Sciences (SPSS) software version 21.0 (IBM Corp., Armonk, NY).

## Results

A total of 100 knees that underwent primary TKA were evaluated. Patient demographics are given in Table 1.

**Table 1:** Distribution of age and gender within the study group

Age and gender	Female	Male	Total	Percentage
<55 years	16	10	26	26%
55-65 years	28	24	52	52%
>65 years	12	10	22	22%
Total	56	44	100	100%

There were no significant differences in age or sex (p-value >0.05).

A comparison between preoperative and postoperative (at the one-year follow-up), Knee Society clinical score is as presented in Table 2.

**Table 2:** Comparing the preoperative and postoperative Knee Society clinical scores between the two groups.

	Patellar resurfacing	Patellar non-resurfacing	p-value
Preoperative Knee Society clinical score			
Sample size	50	50	<0.468
Mean ± SD	25.8 ± 3.89	24.92 ± 2.98	
Postoperative Knee Society clinical score			
Sample size	50	50	<0.001
Mean ± SD	87.92 ± 2.34	85.24 ± 2.22	
p-value	<0.0001	<0.0001	

Both groups showed a significant improvement in postoperative scores compared to preoperative scores (p-value <0.0001 in both groups). However, the intergroup comparison showed significantly higher scores in the patellar resurfacing group (p-value <0.001).

Similarly, on comparison of the Knee Society functional score, significantly higher functional scores were found in the patellar resurfacing group compared to the non-resurfacing group (p-value <0.032) (Table 3).

**Table 3:** Comparing the preoperative and postoperative Knee Society functional scores between the two groups.

	Patellar resurfacing	Patellar non-resurfacing	p-value
Preoperative Knee Society functional score			
Sample size	50	50	<0.853
Mean ± SD	24.8 ± 5.1	25 ± 5	
Postoperative Knee Society functional score			
Sample size	50	50	<0. 032
Mean ± SD	87.4 ± 2.93	85 ± 4.08	
p-value	<0.0001	<0.0001	

There was a significant improvement in postoperative scores at the one-year follow-up in both groups as compared to the preoperative scores (p-value <0.0001 in both groups).

Comparison of VAS scores between both groups showed a significant reduction in postoperative scores in both groups in the study, with a significantly higher postoperative score reduction in the patellar resurfacing group as compared to the non-resurfacing group (Table 4).

**Table 4:** Comparing the preoperative and postoperative visual analogue scale scores between the two groups

	Patellar resurfacing	Patellar non-resurfacing	p-value
Preoperative Visual Analogue Scale			
Sample size	50	50	<0. 725
Mean ± SD	8.2 ± 0.87	8.08 ± 1	
Postoperative Visual Analogue Scale			
Sample size	50	50	<0.047
Mean ± SD	1.96 ± 0.89	2.44 ± 0.77	
p-value	<0.0001	<0.0001	

## Discussion

Total knee arthroplasty has become a widely accepted treatment in orthopaedic surgery to relieve pain and restore function to an arthritic knee [12, 13]. However, opinion is divided among surgeons on the resurfacing of the patella. On one hand, it gives better results in terms of anterior knee pain, which is a cause of major dissatisfaction in patients after TKA; on the other hand, it brings its own set of problems like patellar fractures and maltracking if not executed properly [7, 9].

In our study, a total of 100 patients were randomly allotted to the patellar resurfacing group and the patellar non-resurfacing group. The average preoperative Knee Society clinical score was 25.80 in the patellar resurfacing group, which improved to an average postoperative score of 87.92 with an improvement of 62.12. The average preoperative Knee Society functional score was 24.80 in the patellar resurfacing group, which improved to an average postoperative score of 87.40 with an improvement of 62.60.

The average preoperative Knee Society clinical score was 24.92 in the patellar non-resurfacing group, which improved to an average postoperative score of 85.24 with an improvement of 60.32. The average preoperative Knee Society functional score was 25 in the patellar non-resurfacing group, which improved to an average postoperative score of 85 with an improvement of 60.

Therefore, in our study, we found that TKA in both the patellar resurfacing group and the non-resurfacing group improved the Knee Society clinical and functional scores, and the results of our study are comparable with the results of most other studies [14, 15].

We have also compared the postoperative Knee Society score between the patellar resurfacing group and the patellar non-resurfacing group and found that there is a statistically significant difference in the Knee Society clinical score (p-value of 0.001) and the Knee Society functional score (p-value of 0.032) between the two groups.

The results of our study are comparable with the study done by Waters et al. [5], who performed a prospective, randomised study on 514 knees and reported that overall postoperative knee scores were lower in the non-resurfacing group, and the difference was significant among patients with osteoarthritis (p-value <0.01) and also reported that patients who had a bilateral knee replacement were more likely to prefer the resurfaced side. A study conducted by Schroeder-Boersch et al. [16] also reported better functional outcomes in the knee score in their prospective, randomised control study on 40 knees. Similar results were reported by Pilling et al. [17] in 2012. In their meta-analysis, 1,710 knees underwent patellar resurfacing and 1,755 did not. The functional component of the Knee Society score was significantly higher in the resurfacing group (p = 0.005).

However, the results of our study are not comparable with the study done on 1,421 knees by Shuzhen et al. [14] in 2011. The mean difference in the functional score was not significantly different between the groups.

In our study, the average preoperative VAS score was 8.2 in the patellar resurfacing group, which improved to an average postoperative score of 1.96 with an improvement of 6.24. The average pre-operative VAS score was 8.08 in the patellar non-resurfacing group, which improved to an average postoperative VAS score of 2.44 with an improvement of 5.64.

Therefore, in our study, we found that TKA in both the patellar resurfacing group and the patellar non-resurfacing group improved the VAS score (p-value <0.001). We have compared the postoperative VAS score between both groups and found that there is a statistically significant difference in VAS score between the two groups (p-value = 0.011).

The results of our study are comparable to those done by Wood et al. [3]. Their prospective, randomised study of 220 osteoarthritic knees reported that there was a significantly higher incidence of anterior pain (p = 0.016) in the knees without patellar resurfacing. Also, in 2011, Shuzhen et al. [14] published in their study that the incidence of postoperative anterior knee pain was 12.9% in the patellar resurfacing group and 24.1% in the patellar non-resurfacing group.

However, the results of our study were not comparable with those published by Aunan et al. [15], which included 129 patients in a prospective, randomised control study. This may be because of the smaller sample size in our study. Also, pain as measured by the VAS is not linear, and receptivity can vary [18]. There was no serious complication found in any patient.

The mean period of follow-up in our study was one year (ranging from a maximum of 21 months to a minimum of six months). The limitations of our study were that the sample size was small. Patients were followed up only for one year after surgery. Further studies on larger populations and longer follow-up periods are recommended to truly assess the results of patellar resurfacing.

## Conclusions

This study was undertaken to compare the functional outcomes of patellar resurfacing versus non-resurfacing in patients undergoing TKA. Statistically significant differences were observed in the Knee Society clinical and functional scores between the two groups, and statistically significant improvement was observed in anterior pain assessed with the VAS score between the two groups.

We conclude that patellar resurfacing during TKA provides better clinical and functional outcomes as well as more relief from anterior knee pain as compared to patellar non-resurfacing. However, a large cohort of patients with a longer follow-up is required to conclusively prove or disprove the efficacy of patellar resurfacing.
